# Minimally invasive sacroiliac joint fusion: Primary endpoint results from the prospective, multicenter STACI study

**DOI:** 10.1002/pmrj.70095

**Published:** 2026-01-30

**Authors:** Jacqueline Weisbein, Timothy Davis, Douglas Beall, Jack Smith, Caroline Harstroem, Daniel Kloster, Morteza Rabii, Robyn Capobianco, Ramana Naidu, Michael Harned, Christopher Mallard, the STACI investigators, Jacqueline Weisbein, Christopher Mallard, Michael Harned, Douglas Beall, Dan Nguyen, Jack Smith, Daniel Kloster, Morteza Rabii, Caroline Harstroem, Timothy Davis, Charles Simmons, Denis Patterson, Anne Christopher, John Broadnax, Eric Anderson, Jeffrey Foster, Andrew Trobridge, John Hatheway, Thomas Stauss, Ramana Naidu, Namath Hussain

**Affiliations:** ^1^ Napa Valley Orthopaedics Napa California USA; ^2^ Source Healthcare Santa Monica California USA; ^3^ Clinical Investigations, LLC Edmond Oklahoma USA; ^4^ Argires Marotti Neurosurgical Associates of Lancaster Lancaster Pennsylvania USA; ^5^ Anesis Spine & Pain Care Renton Washington USA; ^6^ Crimson Pain Management Specialists Overland Park Kansas USA; ^7^ SI‐BONE, Inc Santa Clara California USA; ^8^ MarinHealth Spine Institute Larkspur California USA; ^9^ University of Kentucky Lexington Kentucky USA

## Abstract

**Background:**

Chronic sacroiliac joint (SIJ) pain is highly debilitating. Minimally invasive SIJ fusion has become a commonly performed surgical treatment for SIJ pain, with level I evidence showing clinically significant improvements in pain, function, and quality of life and a low adverse event rate. Primarily performed by surgeons, this procedure is increasingly being performed by interventional pain management (IPM) physicians.

**Purpose:**

To evaluate the safety and effectiveness of lateral SIJ fusion performed by IPM physicians, compared with prior studies of similar devices.

**Study Design:**

Prospective, multicenter, single‐arm clinical trial conducted at 15 U.S. sites.

**Patient Sample:**

A total of 112 patients with a diagnosis of SIJ pain, who met study eligibility criteria, underwent lateral SIJ fusion.

**Outcome Measures:**

SIJ pain (numerical rating scale [NRS]), Oswestry Disability Index (ODI), quality of life (Patient‐Reported Outcomes Measurement Information System [PROMIS]‐29), patient satisfaction, and device/procedure‐related adverse events.

**Methods:**

Medical and surgical history and patient‐reported outcomes were assessed at baseline and follow‐up at 1, 3, 6, 12, and 24 months postoperatively. Primary endpoint is change in SIJ pain from baseline to 6 months; statistical evaluation used a noninferiority approach with a 1‐point noninferiority margin compared to prior studies of a similar device. Secondary endpoints included ODI, NRS, PROMIS‐29, device/procedure‐related adverse events, and evidence of fusion via computed tomography scan at 2 years. Twelve‐ and 24‐month follow‐up data collection is ongoing.

**Results:**

Mean (SD) participant age was 64 (14) years and 68% female. No serious or device‐related adverse events occurred. At 6 months, mean SIJ pain decreased 5.2 points (95% confidence interval [CI] 4.7–5.7), meeting the study's primary noninferiority endpoint (*p* < .001 for noninferiority, *p* < .001 for change from baseline). A majority (91%) had a ≥2‐point improvement in SIJ pain. ODI improved by 25.8 points (95% CI 22.2–29.4, *p* < .001 vs. baseline).

**Conclusion:**

Interim trial results support the safety and effectiveness of lateral SIJ fusion performed by IPM physicians. Clinically significant improvements in pain and disability are commensurate with results from surgeon‐performed randomized trials and published literature. Results should be reproduced in randomized trials.

## INTRODUCTION

The sacroiliac joint (SIJ) is structurally a diarthrodial joint but functions as an amphithroidal joint. Its main role is to provide limited mobility and structural stability to the pelvis, aiding in load transfer from the spine to the lower extremities.^1^ Joint degeneration resulting from hyper‐ or hypomobility from trauma, joint laxity, aging, altered biomechanics, arthritis, or increased loading from lumbar spinal fusion can result in SIJ pain. Pain emanating from the SIJ can present in a similar distribution to other spine and hip conditions and is estimated to be responsible for up to 30% of low back pain.^1^

Fusion of the SIJ is a commonly accepted surgical procedure for chronic SIJ pain not responsive to nonsurgical treatment. Initially reported in 1921,^2^ devices specifically designed for minimally invasive SIJ fusion were initial Food and Drug Administration cleared in 2008. As of mid‐2023, 57 studies reporting positive outcomes from minimally invasive SIJ fusion have been published.^3^

Advancements in implant systems and procedural techniques have broadened access of SIJ fusion to nonsurgeon specialties. From 2018 to 2021, nonsurgeon use of SIJ fusion grew 415%. During this time frame, 25% of SIJ fusion procedures were performed by nonsurgeon interventionalists.^4^ Metal devices and allograft implants are available for a variety of technical approaches to accessing the joint, including lateral, posterolateral, posteromedial, dorsal, and posteroinferior. Each technique has its own set of risks and benefits, with various levels and quality of supportive clinical data. Among these, lateral SIJ fusion using an osseointegrative implant stands out for its proven effectiveness and is supported by level I evidence in stabilizing and fusing the joint and clinically significant improvements in pain, function, and quality of life, with a low adverse event rate.^5^, ^6^, ^7^ This technique provides robust fixation and achieves fusion rates of up to 90% by traversing three bony cortices with the implant anchoring in the dense sacral body.^8^, ^9^ Multiple studies, including two randomized controlled trials and a systematic review, support the safety and effectiveness of lateral SIJ fusion with a low rate of implant malposition (0.43%), bleeding requiring intervention (0.04%), or removal for ongoing or recurrent pain (0.06%).^3^, ^5^, ^6^, ^9^, ^10^

Whether SIJ fusion can be safely performed by interventional pain management (IPM) physicians with outcomes similar to previously published studies involving only surgeons is of interest. The objective of the current study is to evaluate outcomes with lateral SIJ fusion when the procedure is performed by IPM physicians.

## METHODS

iFuse TORQ for the Treatment of Sacroiliac Joint Dysfunction (STACI; ClinicalTrials.gov, NCT05870488) is a prospective, multicenter, single‐arm clinical trial conducted at 15 sites in the United States. Principal investigators represent multiple interventional specialties including physical medicine and rehabilitation, interventional radiology, interventional neuroradiology, and anesthesia. Institutional review board approval was received for the study and at each participating clinical site prior to trial initiation. The trial adheres to the principles outlined in the Declaration of Helsinki and International Organization for Standardization 14155. Enrollment was completed in November 2024, and the trial is currently in the follow‐up phase.

Patients with a diagnosis of SIJ pain, confirmed with at least 3/5 positive physical examination maneuvers and a minimum of 50% immediate improvement in SIJ pain on numeric rating scale (NRS) following a diagnostic intraarticular injection, who met all inclusion and no exclusion criteria were eligible to enroll. Inclusion criteria were: a minimum age of 21 years, body mass index ≤35, failure to adequately improve with nonsurgical care (eg, physical therapy, pain medications, SIJ corticosteroid injections and, in some cases, radiofrequency ablation of the SIJ) for at least 6 months, a minimum score of 30% on the Oswestry Disability Index (ODI), and SIJ pain score of at least 5 on a 0–10 NRS (0 = no pain, 10 = worst imaginable pain) at the time of screening.

Patients were excluded if any of the following conditions applied: previous SIJ implant placement on the index side, severe back pain from other causes (ie, lumbar disc degeneration, spinal stenosis, etc.), history of recent (<1 year ago) major trauma to the pelvis, metabolic bone disease, or any other comorbid condition that may interfere with trial participation. Additionally, patients involved in disability litigation or receiving worker's compensation related to back or SIJ pain were excluded. Patients who met all eligibility criteria and provided written informed consent were enrolled in the trial.

### Outcome measures

Baseline assessments consisted of a detailed medical history and patient reported health status questionnaires for functional disability (ODI), SIJ pain (NRS), and quality of life (Patient‐Reported Outcomes Measurement Information System [PROMIS]‐29). ODI, a 10‐question survey that measures the degree of back‐related disability, has been validated for SIJ pain.^11^ PROMIS‐29 is a generic set of health quality measures to evaluate person‐centered physical, mental, and social health independent of therapeutic intervention. Follow‐up visits conducted at 1, 3, 6, 12, and 24 months postoperatively consisted of patient‐reported outcomes and satisfaction with the procedure. Sites were required to report all serious adverse events and nonserious adverse events related to the hip, spine, pelvis, and lower extremities, regardless of their relation to the study device or procedure continuously throughout the trial. A high‐resolution computed tomography scan of the pelvis will be obtained at the final (24‐month) trial visit.

The trial's primary clinical endpoint is the improvement in SIJ pain at 6 months after surgery. Sample size was based on a noninferiority comparison to four prior studies of two previous versions of the study device (Investigation of Sacroiliac Fusion Treatment [INSITE],^5^ Sacroiliac Joint Fusion With iFuse Implant System [SIFI],^12^ and iFuse Implant System Minimally Invasive Arthrodesis [iMIA]^6^ used iFuse implants; Study of Bone Growth in the Sacroiliac Joint After Minimally Invasive Surgery With Titanium Implants [SALLY]^13^ used iFuse 3‐dimensional [3D] implants). In these studies, all of which involved surgeons placing implants, the mean (SD) reduction in SIJ pain was 5 points (2.6 points, 0–10 scale). With 100 participants, the trial has 88% power to detect a change score of at least 4 points from baseline, corresponding to a 5‐point improvement with a 1‐point noninferiority margin.

Secondary endpoints included change in SIJ pain, function, and quality of life over time, and the proportion of participants with an adverse event deemed probably or definitely related to the procedure or device.

### Surgical procedure

Minimally invasive lateral SIJ fusion surgery was performed using 3D printed threaded porous titanium implants (PTI, iFuse TORQ, SI‐BONE). These PTI are self‐drilling and self‐tapping and contain helical flutes designed to self‐harvest bone during placement. The porous surface lattice has pore size and porosity similar to cancellous bone and has been shown to promote bone growth into, onto, and through the implant in an animal model.^14^

The procedure was performed with the patient in the prone position under anesthesia (monitored anesthesia care or general anesthesia, as decided by the anesthesiologist). After appropriate fluoroscopic imaging was obtained, a ~2‐cm skin incision was made, and a guide pin was passed through the soft tissue to the lateral ilium at a superoinferior position just lateral to the S1 neuroforamen. The guide pin was advanced to a depth just lateral to the S1 neuroforamen, verifying the trajectory throughout using inlet and outlet views on fluoroscopy. After the soft tissue was dilated and pin depth measured, an appropriately sized PTI was inserted. A parallel pin guide was then placed over the first guide pin and used to help position subsequent guide pins for additional PTIs. Identical steps were used to place the additional PTI. Two or three PTIs were placed on each side, depending on the patient's anatomy. Final PTI position was verified on pelvic inlet, outlet, and lateral views (Figure 2). Perioperative measures collected included estimated blood loss, operating time, devices used, and adverse events.

### Statistical Analysis

All statistical analyses were performed using R.^15^ Continuous variables are summarized as means and SD. The primary endpoint, the improvement from baseline to 6 months in SIJ pain score, was evaluated against the primary noninferiority hypothesis using a one‐sided Student's *t*‐test. Additional analyses of continuous variables, for example, in quality of life scores at all postoperative time points, were evaluated using repeated measures analysis of variance. To take into account differences in pain locations and treatments and to preserve statistical independence, SIJ pain scores were collated as follows: for participants with unilateral pain who underwent unilateral treatment, the SIJ pain score on the treated side was used. For participants with bilateral SIJ pain at baseline who underwent staged bilateral SIJ fusion, pain scores for the index side were used until the contralateral side was treated, after which pain scores were averaged across the two sides. For participants with bilateral pain at baseline but who underwent unilateral treatment only, pain scores from the index side only were used. No adjustment for ODI scores was used.

## RESULTS

**FIGURE 1:**
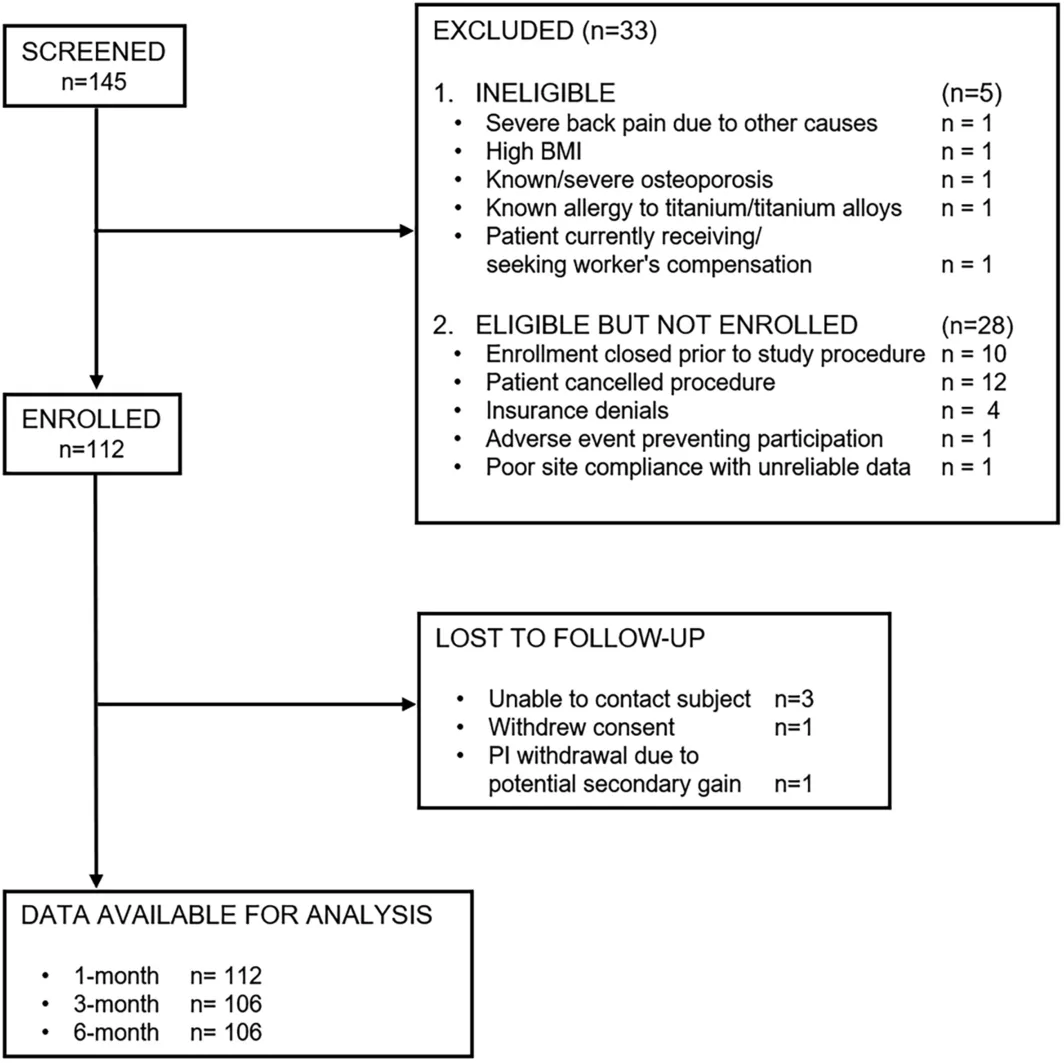
Patient flow diagram. BMI, body mass index; PI, principal investigator.

A total of 112 participants were enrolled across 15 sites (Figure 1). Mean participant age (SD, range) at trial entry was 63.8 years (14.1, 25–89), 67.9% were female, and the mean body mass index was 29.5 (5.1). At baseline, 43.8% of participants were taking opioid pain medication (Table 1).

**TABLE 1:** Demographics.

	*n* = 112
Age (y), mean (SD)	63.8 (14.1)
Female gender, *N* (%)	76 (67.9%)
Body mass index, kg/m^2^, mean (SD)	29.5 (5.1)
Smoking, *N* (%)	
Current	7 (6.2%)
Former	46 (41.1%)
Nonsmoker	57 (50.9%)
Other tobacco use	2 (1.8%)
Pain duration (y), mean (SD)	7.0 (9.6)
ODI score, mean (SD)	52.1 (13.8)
SIJ pain score, mean (SD)	7.5 (1.4)
Taking opioid medication, N (%)	49 (43.8%)

Abbreviations: ODI, Oswestry Disability Index; SIJ, sacroiliac joint.

**FIGURE 2:**
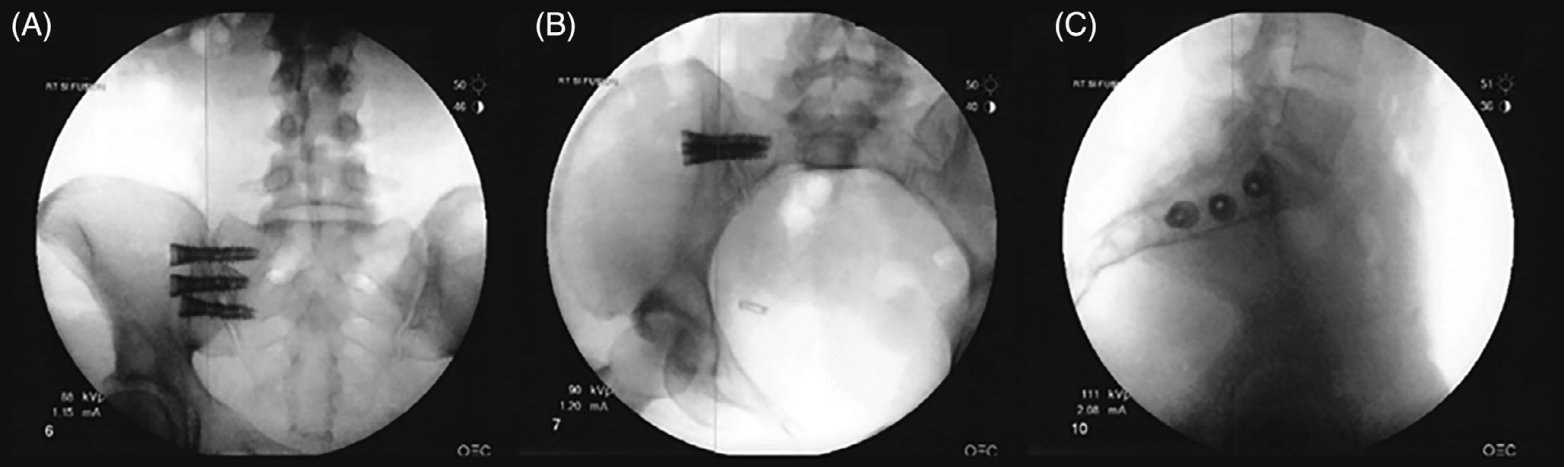
Pelvic outlet (A), inlet (b), and lateral (C) images.

A total of 131 procedures were performed in 112 participants. Five participants with bilateral pain underwent staged bilateral surgery, with a mean time to second‐side surgery of 42 days (range 21–93 days). An additional 13 participants with bilateral pain initially chose to treat only the more painful side; they later underwent surgery on the contralateral side at an average of 176 days (range 75–287) after the initial procedure. One patient developed new onset contralateral SIJ pain and underwent contralateral SIJ fusion at day 269.

**FIGURE 3:**
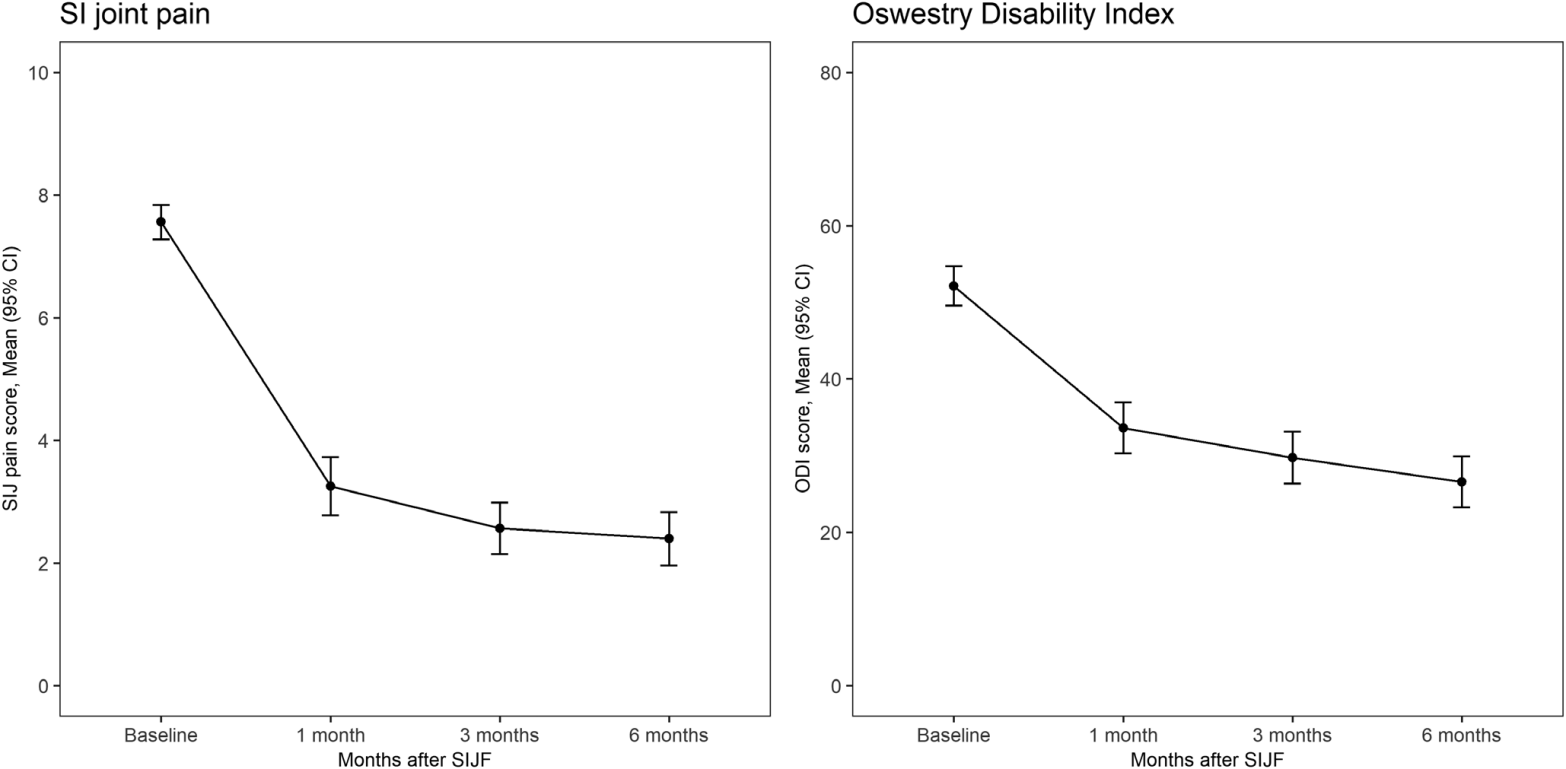
SIJ pain and ODI scores by visit. CI, confidence interval; ODI, Oswestry Disability Index; SI, sacroiliac; SIJ, sacroiliac joint; SIJF, sacroiliac joint fusion.

**FIGURE 4:**
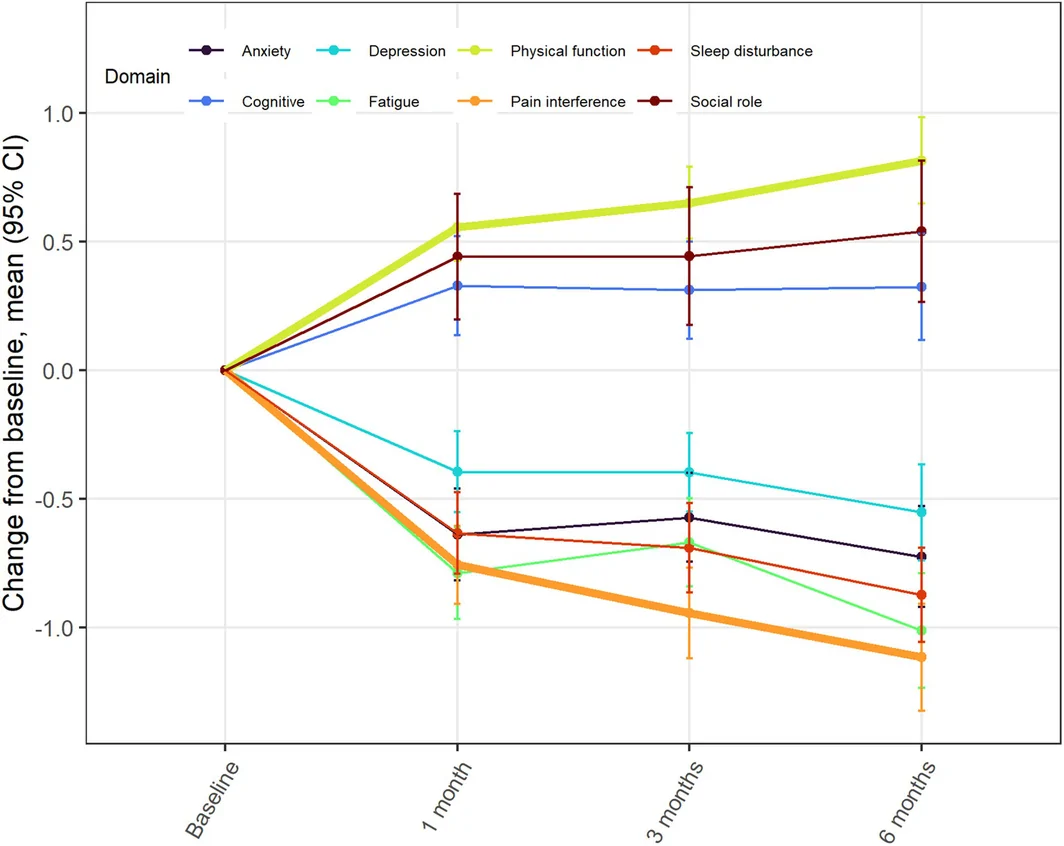
Significant improvements were observed across all domains of PROMIS‐29. CI, confidence interval; PROMIS, Patient‐Reported Outcomes Measurement Information System.

**FIGURE 5:**
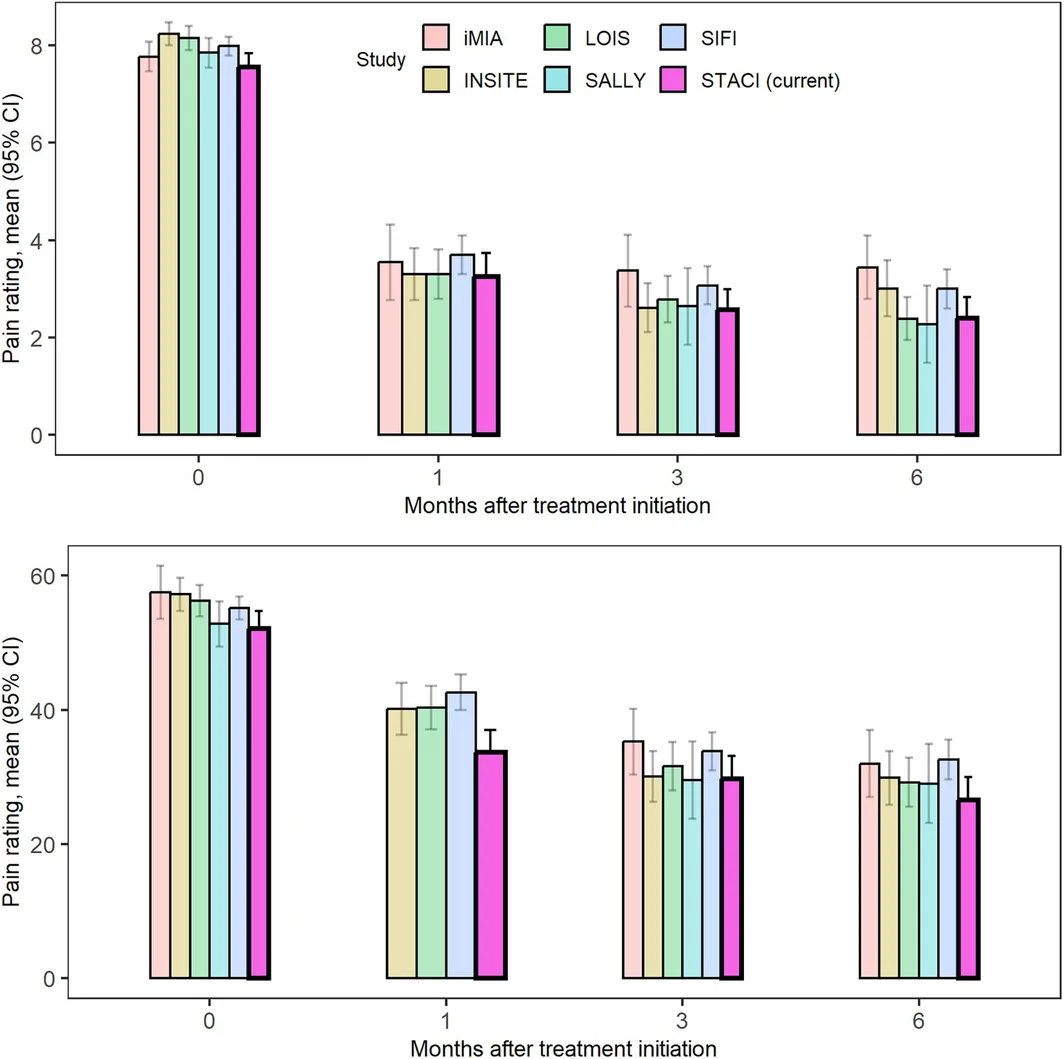
SIJ pain and ODI values compare favorably with previous studies of lateral SIJ fusion. iMIA, iFuse Implant System Minimally Invasive Arthrodesis; INSITE, Investigation of Sacroiliac Fusion Treatment; LOIS, Long‐Term Follow‐Up in INSITE/SIFI; ODI, Oswestry Disability Index; SALLY, Study of Bone Growth in the Sacroiliac Joint After Minimally Invasive Surgery With Titanium Implants; SIFI, Sacroiliac Joint Fusion With iFuse Implant System; SIJ, sacroiliac joint; STACI, iFuse TORQ for the Treatment of Sacroiliac Joint Dysfunction.

All trial procedures were performed in the outpatient setting. Mean (SD) procedure time was 43.9 (15.8) minutes and blood loss was minimal, 12.3 (12.1) mL (Table 2). No intraoperative adverse events or procedural complications occurred.

**TABLE 2:** Procedure characteristics (first procedure only).

	*n* = 112
Est. blood loss, mean (SD)	12.4 mL (12.1)
Implants used	
2 per side	15
3 per side	97
Anesthesia type	
Monitored anesthesia care	29 (25.9%)
General	83 (74.1%)

During follow‐up, four events were deemed possibly, probably, or definitely related to PTI or placement of PTI (Table 3). One participant had ipsilateral groin pain approximately 6 months after index surgery; there were radiographic signs of hip osteoarthritis but no issues related to PTI. One participant experienced wound dehiscence at day 26. One participant had sudden onset ipsilateral buttock pain at day 40. The participant was withdrawn from the trial after the 1‐month follow‐up visit due to a complex social situation. The participant underwent replacement of the inferior‐most device due to ongoing buttock pain. There was no implant lucency on computed tomography. Thereafter, SIJ pain was greatly improved. One participant had transient increase in SIJ pain after a contralateral SIJ fusion with PTI. Additional adverse events deemed possibly, probably, or definitely related to index surgery included: skin reaction to adhesive (*n* = 1), hematoma (*n* = 1), wound infection (*n* = 1), lumbar radiculopathy (*n* = 1), and ipsilateral groin pain at month 12 (*n* = 1) (Table 3). In this last case, hip anesthetic injection produced transient complete pain relief. Further information on this case is not available. Sixteen additional adverse events were reported in 15 participants (Table 4). The most commonly reported events were minor falls (*n* = 4) and hip labral tears (*n* = 2).

**TABLE 3:** Adverse events possibly, probably, or definitely related to PTI or PTI placement procedure and events related to surgery. One event of each type occurred during follow‐up.

Event	Days from surgery to adverse event
Related to PTI or PTI placement	
Ipsilateral groin pain, possible hip osteoarthritis	176
Sudden onset ipsilateral buttock pain	40
Wound dehiscence	26
Sacroiliac joint pain	159
Related to surgery	
Skin reaction to adhesive	1
Hematoma	0
Wound infection	13
Lumbar radiculopathy	14
Ipsilateral thigh muscle spasm	5

Abbreviation: PTI, porous titanium implant.

**TABLE 4:** Adverse events not related to PTI, PTI placement procedure or surgery.

Event	*N*	Days from surgery to adverse event	Range^a^
Chronic pancreatitis	1	169	‐
Cluneal neuralgia	1	36	‐
Facet arthritis	1	95	‐
Fall	4	72	[20–123]
Flank tenderness	1	26	‐
Hip labrum tear	2	60	[32–87]
Low back pain	1	202	‐
Piriformis syndrome	1	31	‐
Spinal stenosis	1	76	‐
Thoracic pain	1	187	‐
Trochanteric bursitis	1	21	‐
Worsening of bladder fistula	1	5	‐

^a^ If more than one event.

Abbreviation: PTI, porous titanium implant.

Significant improvements in pain and function were observed at the 1‐month visit, with continued improvement by the 6‐month visit (Figure 3, Table 5). SIJ pain decreased from a mean (SD) of 7.6 (1.5) at baseline to 2.4 (2.3) at 6 months, a 5.2‐point improvement (95% confidence interval [CI] 4.7–5.7; *p* < .0001). The change score met the trial's noninferiority primary endpoint hypothesis (*p* < .001). Further, 90.6% of participants achieved a clinically meaningful reduction in pain score (≥2 points), with 71.7% experiencing a reduction of 4 points or more.^16^ Similarly, mean (SD) ODI scores improved from 52.1 (13.8) at baseline to 26.8 (16.6) at 6 months, an average improvement of 25.8 points (95% CI 22.2–29.4, Figure 3; *p* < .001). A clinically meaningful reduction of ≥13 points was observed in 76.2% of participants.^11^ At 6 months, participant satisfaction was high; 75.5% were very satisfied and 90.6% were somewhat or very satisfied. Improvements were observed across all domains of PROMIS‐29. Clinically meaningful (≥0.5) and statistically significant (*p* < .001) changes were observed on pain interference and physical function (Figure 4).^17^

**TABLE 5:** Patient‐reported outcomes.

	Visit	*N*	Mean (SD)	Change from baseline	*p* value*
SIJ pain	Baseline	112	7.6 (1.5)	‐	‐
	1 mo	109	3.3 (2.5)	−4.4 (2.9)	<.0001
	3 mo	106	2.6 (2.2)	−5.0 (2.6)	<.0001
	6 mo	106	2.4 (2.3)	−5.2 (2.7)	<.0001
Oswestry Disability Index	Baseline	112	52.1 (13.7)	‐	‐
	1 mo	109	33.6 (17.8)	−18.9 (18.4)	<.0001
	3 mo	105	29.7 (17.6)	−22.9 (18.1)	<.0001
	6 mo	105	26.6 (17.3)	−25.8 (18.7)	<.0001
PROMIS‐29					
Anxiety	Baseline	108	0.45 (1.01)	‐	‐
	1 mo	107	−0.17 (0.90)	−0.64 (0.92)	<.0001
	3 mo	105	−0.11 (0.98)	−0.57 (0.87)	<.0001
	6 mo	104	−0.25 (0.90)	−0.72 (0.99)	<.0001
Cognitive	Baseline	108	−0.11 (0.68)	‐	‐
	1 mo	107	0.21 (0.81)	0.32 (0.99)	.0013
	3 mo	105	0.22 (0.85)	0.31 (0.96)	.0017
	6 mo	104	0.25 (0.88)	0.33 (1.05)	.0024
Depression	Baseline	108	0.18 (0.94)	‐	‐
	1 mo	107	−0.18 (0.81)	−0.39 (0.8)	<.0001
	3 mon	105	−0.19 (0.90)	−0.39 (0.78)	<.0001
	6 mo	104	−0.32 (0.86)	−0.55 (0.94)	<.0001
Fatigue	Baseline	108	0.98 (0.94)	‐	‐
	1 mo	107	0.23 (0.96)	−0.78 (0.92)	<.0001
	3 mo	105	0.32 (1.01)	−0.67 (0.87)	<.0001
	6 mo	104	0.02 (1.03)	−1.00 (1.13)	<.0001
Pain interference	Baseline	108	1.74 (0.57)	‐	‐
	1 mo	107	1.00 (0.76)	−0.75 (0.78)	<.0001
	3 mo	105	0.82 (0.92)	−0.94 (0.9)	<.0001
	6 mo	104	0.66 (0.96)	−1.11 (1.05)	<.0001
Physical function	Baseline	108	−1.65 (0.48)	‐	‐
	1 mo	107	−1.15 (0.71)	0.54 (0.68)	<.0001
	3 mo	105	−1.04 (0.77)	0.65 (0.71)	<.0001
	6 mo	105	−0.88 (0.78)	0.81 (0.85)	<.0001
Sleep disturbance	Baseline	108	0.80 (0.95)	‐	‐
	1 mo	107	0.19 (0.89)	−0.63 (0.81)	<.0001
	3 mo	105	0.15 (0.97)	−0.69 (0.89)	<.0001
	6 mo	104	−0.03 (0.96)	−0.87 (0.93)	<.0001
Social role	Baseline	108	−0.5 (1.1)	‐	‐
	1 mo	107	−0.07 (0.89)	0.43 (1.26)	.0008
	3 mo	105	−0.11 (0.94)	0.46 (1.37)	.0010
	6 mo	105	0.01 (0.95)	0.56 (1.41)	.0001

* *p* value from Student's *t*‐test.

Abbreviations: PROMIS, Patient‐Reported Outcomes Measurement Information System; SIJ, sacroiliac joint.

## DISCUSSION

Our trial achieved its objectives and primary endpoint. Six‐month results show that lateral SIJ fusion can be safely and effectively performed by IPM physicians. The trial met its predetermined primary endpoint, a noninferiority comparison to prior trials^5^, ^6^, ^12^, ^13^ of similar devices. Participant satisfaction with the procedure was high. There were no cases of excessive bleeding, and the three procedure‐related adverse events were wound related.

Chronic low back pain is often multifactorial, involving multiple pain generators beyond the SIJ, including preexisting spinal pathology, altered gait mechanics, nerve‐related dysfunction, and musculoskeletal imbalances. This is reflected in the types of nonprocedure‐related adverse events reported in the trial, which included hip labrum issues, falls, and exacerbation of spinal stenosis symptoms.

The pain reduction and functional improvement observed in our trial are considerable given the complex patient population. On average, SIJ pain decreased by 5 points, a 72% decrease from baseline and twice the value reported for substantial clinical benefit (2.5‐point change) for lumbar spine surgery.^18^ More than 70% of participants experienced a decrease in SIJ pain by 4 points. ODI decreased by 26 points, a 50% improvement from baseline, twice the reported minimal clinically important difference and greater than the 18 points identified as substantial clinical benefit for patients undergoing lumbar spine surgery.^11^, ^18^ Our results, although similar to those reported in a large meta‐analysis of SIJ fusion as performed by surgeons,^2^ differ from those reported in a sham‐controlled trial,^19^ which, in addition to having a sham surgical control, showed improvements in SIJ pain that were smaller than observed in our study.

The rate of adverse events in our trial was similar to those studies for previous versions of the study device. Specifically, we observed 1 case (0.9%) of bleeding (a hematoma, not requiring surgical management); in prior studies of similar devices,^5^, ^6^, ^12^, ^13^ 2 of 377 (0.5%) participants had bleeding requiring interventions. Wound dehiscence or wound infection occurred in 2 participants (1.8%) in our trial and 6 (1.6%) in prior studies.

Importantly, outcomes in our trial, in which all procedures were performed by IPM physicians, compared favorably with surgeon‐published literature.^6^, ^20^, ^21^ In a recent systematic review and meta‐analysis, the average improvements in pain (NRS) and function (ODI) were 4.8 and 25.9, respectively.^3^ The reduction in SIJ pain scores in our trial followed a trajectory similar to those observed in previous prospective trials of prior versions of the device from the same manufacturer (Figure 5). These previous studies evaluated SIJ fusion using an impacted triangular PTI, whereas the procedure in the current trial was conducted by IPM physicians using a 3D‐printed, self‐tapping, threaded implant. Notably, there were no instances of excessive bleeding and to date, no implant revisions have occurred in our trial.

Previous research across various medical specialties has highlighted strong correlations between surgeon case volume and fellowship training with improved postoperative outcomes.^22^, ^23^ However, many procedures once performed exclusively by surgeons are now routinely undertaken by interventional physicians, with limited evidence suggesting that one specialty consistently outperforms the other. For instance, studies on carotid artery stenting found equivalent perioperative safety outcomes between surgeons and interventionalists.^24^ Among percutaneous procedures, vertebral augmentation procedures (eg, kyphoplasty) are relatively evenly distributed between surgeons and interventionalists, with long‐term (5‐year) outcomes and reoperation rates showing no statistically significant differences between groups.^19^ Furthermore, research indicates that surgeons who perform a higher volume of specific procedures consistently achieve better outcomes and lower revision rates than those with lower procedural volumes.^25^, ^26^ Therefore, it appears that physician procedural volume, more so than physician specialty, is a potential factor influencing operative complications and patient outcomes.

### Limitations

The primary limitation of this trial is the absence of a concurrent group of participants undergoing the same procedure performed by physicians formally trained in surgery. Prior published information on surgeon‐derived outcomes for this procedure is ample (four prior prospective trials using prior versions of the study device as well as a published meta‐analysis,^3^ which included >40 studies of outcomes from SIJ fusion procedures performed by surgeons). The observed improvements in pain scores and ODI in our study were very similar to prior studies (Figure 5). Combined with the observed low rate of device‐ and procedure‐related adverse events, our data support the safety and effectiveness of the procedure when performed by trained interventionalists. The other main limitation of our study is the relatively subjective nature of pain and disability scores.

## CONCLUSION

Results from this trial provide evidence to support the safety and effectiveness of lateral SIJ fusion when performed by IPM physicians. The significant improvements in pain and disability are consistent with previous published studies in which procedures were performed by surgeons. Results should be reproduced in randomized trials.

## FUNDING INFORMATION

This study was sponsored by SI‐BONE, Inc. No funds were received in support of this work.

## Disclosure

Jacqueline Weisbein reports consulting fees from SI‐Bone and Abbott; and payment or honoraria for lectures/presentations from AbbVie and Vertex. Christopher Mallard reports grants or contracts from Medtronic; and consulting fees from Johnson & Johnson and SI‐Bone. Douglas Beall reports consulting fees from SI‐Bone; payment or honoraria for lectures, presentations, speakers bureaus, manuscript writing or educational events from Medtronic, ReGelTec, Nanofuse, Talosix, Spinal Simplicity, Spark Biomedical, Smart Soft, Tissue Tech, Bronx Medical, Thermaquil, Vivex, Genesys, SetBone Medical, Amber Implants, Cerapedics, SpinaFX, SI‐Bone, AIS Healthcare, Maho MT (US), and US Healthcare; and support for attending meetings and/or travel from Stryker, Medtronic, Boston Scientific, Merit, Avanos, Piramal, Genesys, Eliquence, AIS Healthcare, and Amgen. Robyn Capobianco reports stock or stock options in SI‐Bone. Timothy Davis reports consulting fees from SI‐Bone, Medtronic, Boston Scientific, Spine BioPharma, Spinal Simplicity, VIVEX, DiscGenics, Saluda, Abbott, Kolon TissueGene, and Tenex Health; payment or honoraria for lectures, presentations, speakers bureaus, manuscript writing or educational events from Medtronic, VIVEX, Mesoblast, SI‐Bone, Abbott, Spine BioPharma, Spinal Solutions, NatureCell, DiscGenics, Kolon TissueGene, and Boston Scientific; and support for attending meetings from SI‐Bone. Jack Smith reports consulting fees and payment or honoraria for lectures, presentations, speaker's bureaus, manuscript writing or educational events from SI‐Bone. Daniel Kloster reports payment or honoraria for lectures, presentations, speakers bureaus, manuscript writing or educational events from Abbott, Spinal Simplicity, NeuroOne, Boston Scientific, and Nevro; and stock or stock options from NeuroOne, Spinal Simplicity, and inFormed Consent. Ramana Naidu reports participation on a data safety monitoring board for SI‐Bone. Morteza Rabii reports grants or contracts from Abbott; consulting fees from Abbott Neuromodulation, SI‐Bone, Spinal Simplicity, and inFormed Consent. Caroline Harstroem reports grants or contracts and research support from SI‐Bone related to the STACI study, including payments to Research/Consulting LLC. The remainder of the authors have no conflicts of interest to disclose.

## Data Availability

Research data are not shared.

## APPENDIX A STACI INVESTIGATORS

Jacqueline Weisbein, Christopher Mallard, Michael Harned, Douglas Beall, Dan Nguyen, Jack Smith, Daniel Kloster, Morteza Rabii, Caroline Harstroem, Timothy Davis, Charles Simmons, Denis Patterson, Anne Christopher, John Broadnax, Eric Anderson, Jeffrey Foster, Andrew Trobridge, John Hatheway, Thomas Stauss, Ramana Naidu*, and Namath Hussain.* *Data Monitoring Committee.

## References

[1] Sembrano JN , Polly DW . How often is low back pain not coming from the back? Spine. 2009;34:E27‐E32. doi:10.1097/BRS.0b013e31818b8882 19127145

[2] Smith‐Petersen MN . Arthrodesis of the sacroiliac joint. A new method of approach. J Bone Joint Surg. 1921;3:400‐405.

[3] Whang PG , Patel V , Duhon B , et al. Minimally invasive SI joint fusion procedures for chronic SI joint pain: systematic review and meta‐analysis of safety and efficacy. Int J Spine Surg. 2023;17:8543. doi:10.14444/8543 PMC10753354 37798076

[4] Dada A , Dalle Ore C , Mummaneni PV , et al. The exponential growth of nonsurgeons performing fusions for low‐back pain. J Neurosurg Spine. 2024;41:1‐8. doi:10.3171/2024.6.SPINE24311 39332029

[5] Polly DW , Swofford J , Whang PG , et al. Two‐year outcomes from a randomized controlled trial of minimally invasive sacroiliac joint fusion vs. non‐surgical Management for Sacroiliac Joint Dysfunction. Int J Spine Surg. 2016;10:Article 28:1‐22. doi:10.14444/3028 27652199 PMC5027818

[6] Dengler J , Kools D , Pflugmacher R , et al. Randomized trial of sacroiliac joint arthrodesis compared with conservative management for chronic low back pain attributed to the sacroiliac joint. J Bone Joint Surg Am. 2019;101:400‐411. doi:10.2106/JBJS.18.00022 30845034 PMC6467578

[7] Darr E , Meyer SC , Whang PG , et al. Long‐term prospective outcomes after minimally invasive trans‐iliac sacroiliac joint fusion using triangular titanium implants. Med Devices Evid Res. 2018;11:113‐121. doi:10.2147/MDER.S160989 PMC5898880 29674852

[8] Bruna‐Rosso C , Arnoux P‐J , Bianco R‐J , Godio‐Raboutet Y , Fradet L , Aubin C‐É . Finite element analysis of sacroiliac joint fixation under compression loads. Int J Spine Surg. 2016;10:16. doi:10.14444/3016 27441174 PMC4943166

[9] Whang PG , Darr E , Meyer SC , et al. Long‐term prospective clinical and radiographic outcomes after minimally invasive lateral transiliac sacroiliac joint fusion using triangular titanium implants. Med Devices (Auckl). 2019;12:411‐422 [Epub 2019 Sep 26]. doi:10.2147/MDER.S219862 31576181 PMC6769032

[10] Polly DW . Minimally invasive sacroiliac joint fusion vs. conservative management for chronic sacroiliac joint pain. J Spine Surg (Hong Kong). 2019;5:381‐383. doi:10.21037/jss.2019.06.10 PMC6787368 31663051

[11] Copay AG , Cher DJ . Is the Oswestry Disability Index a valid measure of response to sacroiliac joint treatment? Qual Life Res. 2016;25:283‐292 [Epub 2015 Aug 6]. doi:10.1007/s11136-015-1095-3 26245709 PMC4722083

[12] Duhon BS , Cher DJ , Wine KD , Kovalsky DA , Lockstadt H . Triangular titanium implants for minimally invasive sacroiliac joint fusion: a prospective study. Glob Spine J. 2016;6:257‐269. doi:10.1055/s-0035-1562912 PMC4836932 27099817

[13] Patel V , Meyer SC , Kovalsky D , et al. Prospective trial of sacroiliac joint fusion using 3D‐printed triangular titanium implants: five‐year follow‐up. Spine. 2025;50:620‐627. doi:10.1097/BRS.0000000000005170 39344079 PMC11970590

[14] Polly DW , Gardner MJ , Wills D , et al. Osseointegration and fixation opportunities of sacroiliac instrumentation: a preclinical evaluation in a large animal model. J Spine Surg Hong Kong. 2024;10:616‐626. doi:10.21037/jss-24-67 PMC11732319 39816774

[15] R Core Team . R: A Language and Environment for Statistical Computing. 2014.

[16] Childs JD , Piva SR , Fritz JM . Responsiveness of the numeric pain rating scale in patients with low back pain. Spine. 2005;30:1331‐1334. 15928561 10.1097/01.brs.0000164099.92112.29

[17] Khutok K , Janwantanakul P , Jensen MP , Kanlayanaphotporn R . Responsiveness of the PROMIS‐29 scales in individuals with chronic low Back pain. Spine. 2021;46:107‐113. doi:10.1097/BRS.0000000000003724 33347091

[18] Glassman SD , Copay AG , Berven SH , Polly DW , Subach BR , Carreon LY . Defining substantial clinical benefit following lumbar spine arthrodesis. J Bone Joint Surg Am. 2008;90:1839‐1847. doi:10.2106/JBJS.G.01095 18762642

[19] Hogan WB , Philips A , Alsoof D , et al. Kyphoplasty and vertebroplasty performed by surgeons versus nonsurgeons: trends in procedure rates, complications, and revisions. World Neurosurg. 2022;164:e518‐e524. doi:10.1016/j.wneu.2022.05.004 35552034

[20] Duhon BS , Cher DJ , Wine KD , Lockstadt H , Kovalsky D , Soo C‐L . Safety and 6‐month effectiveness of minimally invasive sacroiliac joint fusion: a prospective study. Med Devices (Auckl). 2013;6:219‐229. doi:10.2147/MDER.S55197 24363562 PMC3865972

[21] Whang PG , Cher D , Polly D , et al. Sacroiliac joint fusion using triangular titanium implants vs. non‐surgical management: six‐month outcomes from a prospective randomized controlled trial. Int J Spine Surg. 2015;9:6. doi:10.14444/2006 25785242 PMC4360612

[22] Birkmeyer JD , Stukel TA , Siewers AE , Goodney PP , Wennberg DE , Lucas FL . Surgeon volume and operative mortality in the United States. N Engl J Med. 2003;349:2117‐2127. doi:10.1056/NEJMsa035205 14645640

[23] Chowdhury MM , Dagash H , Pierro A . A systematic review of the impact of volume of surgery and specialization on patient outcome. Br J Surg. 2007;94:145‐161. doi:10.1002/bjs.5714 17256810

[24] Steppacher R , Csikesz N , Eslami M , Arous E , Messina L , Schanzer A . An analysis of carotid artery stenting procedures performed in New York and Florida (2005‐2006): procedure indication, stroke rate, and mortality rate are equivalent for vascular surgeons and non‐vascular surgeons. J Vasc Surg. 2009;49:1379‐1385. doi:10.1016/j.jvs.2009.02.233 19497495

[25] Brennand EA , Quan H . Evaluation of the effect of Surgeon's operative volume and specialty on likelihood of revision after mesh midurethral sling placement. Obstet Gynecol. 2019;133:1099‐1108. doi:10.1097/AOG.0000000000003275 31135723 PMC6553521

[26] Cossec CL , Colas S , Zureik M . Relative impact of hospital and surgeon procedure volumes on primary total hip arthroplasty revision: a nationwide cohort study in France. Arthroplasty Today. 2017;3:176‐182. doi:10.1016/j.artd.2017.03.010 28913403 PMC5585819

